# Origin and evolution of the *Rax* homeobox gene by comprehensive evolutionary analysis

**DOI:** 10.1002/2211-5463.12832

**Published:** 2020-03-19

**Authors:** Tetsuo Kon, Takahisa Furukawa

**Affiliations:** ^1^ Laboratory for Molecular and Developmental Biology Institute for Protein Research Osaka University Suita Japan

**Keywords:** eye, homeobox gene, molecular evolution, Pax6, Rax, retina

## Abstract

Rax is one of the key transcription factors crucial for vertebrate eye development. In this study, we conducted comprehensive evolutionary analysis of Rax. We found that Bilateria and Cnidaria possess *Rax,* but Placozoa, Porifera, and Ctenophora do not, implying that the origin of the *Rax* gene dates back to the common ancestor of Cnidaria and Bilateria. The results of molecular phylogenetic and synteny analyses on *Rax* loci between jawed and jawless vertebrates indicate that segmental duplication of the *Rax* locus occurred in an early common ancestor of jawed vertebrates, resulting in two *Rax* paralogs in jawed vertebrates, *Rax* and *Rax2.* By analyzing 86 mammalian genomes from all four major groups of mammals, we found that at least five independent *Rax2* gene loss events occurred in mammals. This study may provide novel insights into the evolution of the eye.

## Graphical abstract

Evolution of RAX transcription factor, crucial for eye development, was comprehensively analyzed. Our analyses suggest that *Rax* appeared in the common ancestor of Cnidaria and Bilateria. Jawed vertebrate *Rax* and *Rax2* might originate from segmental duplication of the ancient *Rax* locus. At least five independent *Rax2* gene loss events occurred in mammals. This study provides novel insights into eye evolution.

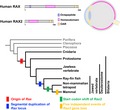

**Abbreviations**
JTT: Jones–Taylor–Thornton model
Ka/Ks: nonsynonymous‐to‐synonymous substitution ratio
LG: Le–Gascuel model
WAG: Whelan and Goldman model
WGD: whole‐genome duplication

Acquiring visual information from the external environment is critical for animal survival. Over the course of evolution, animals have developed different kinds of eyes, from eyespots to complex refractive and compound eyes, which allow them to respond to light stimulus [1]. Notably, vertebrates have developed camera eyes, in which the retina receives the visual input [1].

The homeobox gene superfamily encodes transcription factors with diverse functional roles [2]. For DNA recognition, these transcription factors share a 60‐amino‐acid homeodomain, which comprises a helix‐turn‐helix structure, similar to the one found in prokaryotic gene regulatory proteins [3]. Since animals, plants, and fungi possess homeobox genes, the origin of such genes preceded the divergence of these kingdoms [4]. Among these kingdoms, animal homeobox genes are the most diverse due to extensive gene duplication in the early eumetazoan lineage [5].

We previously identified the retina and anterior neural fold homeobox (*Rax*, also known as *Rx*) gene, which plays critical roles in the eye and forebrain development of vertebrate species [6, 7, 8]. Vertebrate *Rax* is composed of an N‐terminal octapeptide, a paired‐type homeobox, and a C‐terminal OAR motif [6]. In the early mouse embryo, *Rax* is expressed in the anterior neural fold [6]. Subsequently, its expression is limited to the embryonic diencephalon region, which develops into the retina and pineal gland [6]. *Rax*‐null mouse embryos do not form optic vesicles and exhibit the reduction of brain structures [8]. Likewise, mutations of the *RAX* gene were reported in human microphthalmia patients [9, 10]. In the retina, *Rax* plays an essential role in cell fate determination and maturation of photoreceptor cells [11, 12]. It has been shown that *Pax6*, another homeobox gene, plays an essential role in eye development [13]. It should be noted that, in *Pax6*‐null mouse embryos, optic vesicles are formed and the *Rax* expression is unaffected, but cell proliferation in optic vesicles is severely impaired, resulting in a defective eye structure at later developmental stages. On the other hand, the cornea and lens are not formed in *Pax6*‐null mouse embryos [14]. Thus, Rax is one of the critical transcription factors functioning at the initial stage of eye development, but acts independently of Pax6 [15, 16].

As a result of recent advances in DNA sequencing and computation, whole‐genome sequencing has become widely available [17]. To date, 2618 animal genomes including those of 1171 invertebrates have been sequenced, according to the NCBI Assembly database [18]. Since invertebrates diverged from vertebrates more than 500 million years ago [19], the genome sequences of invertebrates provide us with an unique opportunity to study the origin and evolution of genes [20]. Previous studies on the molecular evolution of *Rax* focused primarily on vertebrates [21] or on Bilateria, Cnidaria, and Placozoa [22, 23]. This may be due to the fact that the number of genomic sequences available when these studies were conducted was very limited compared to those currently available. Therefore, to investigate the origin and molecular evolution of *Rax* and to gain insights into the evolution of the eye, we conducted a comprehensive evolutionary analysis of *Rax*. To do so, we analyzed the abundant number of currently available genome sequences, including Ctenophora and Porifera in addition to Bilateria, Cnidaria, and Placozoa.

## Materials and methods

### Collection of *Rax* orthologs

To identify *Rax* orthologs, we downloaded NCBI Gnomon gene models of various species (https://www.ncbi.nlm.nih.gov/genome/annotation_euk/gnomon/). Gnomon gene models were constructed using comprehensive gene predictions with a combination of homology searching and *ab initio* modeling. For every species analyzed, we obtained protein‐coding sequences from genome sequences based on the corresponding NCBI Gnomon gene models. We obtained complete protein sets by translating these protein‐coding sequences. To identify putative Rax ortholog sequences, we performed blastp against every complete set of protein sequences using the Rax protein sequences for human (NP_038463.2), octopus (XP_014777656.1), and *Pocillopora damicornis* (XP_027036745.1) as query sequences with *E*‐values < 1e−10. In cases where *Rax* was not identified in the complete set of protein sequences in a species, we performed tblastn against its genome sequence using the above three query sequences with *E*‐values < 1e−10. All protein sequences for putative Rax orthologs were subjected to a blastp search against all human protein sequences to confirm their orthologous relationships with human RAX. To identify protein‐coding sequences of *Rax* from transcriptomic data, we downloaded publicly available RNA‐seq raw reads from the NCBI SRA database [24] and performed *de novo* transcriptome assembly using Trinity under default settings [25]. We searched for *Rax* putative protein‐coding sequences in the Trinity contigs using tblastn as described above. The protein sequence of *Meara stichopi* Rax (AVK72338.1) [26] was obtained from the NCBI nucleotide database. In every Rax ortholog, regions of the octapeptide, homeodomain, and OAR motif were defined with reference to human RAX. The complete list of accession numbers for the taxon names, genome assemblies, and *Rax* orthologs is provided in Tables 1 and 2. Domain organizations of genes or proteins were illustrated by Illustrator for Biological Sequences [27].

**Table 1:** *Rax* and *Rax2* orthologs in various animal species.

Group	Taxon name	Genome or transcriptome	Reference ID	Genome assembly level	*Rax*	*Rax2*
Porifera	*Amphimedon queenslandica*	Genome	GCA_000090795.1	Scaffold	–	–
Porifera	*Aplysina aerophoba*	Genome	GCA_900275595.1	Contig	–	–
Porifera	*Sycon ciliatum*	Transcriptome	ERR466755	n.a.	–	–
Ctenophora	*Pleurobrachia bachei*	Genome	GCA_000695325.1	Scaffold	–	–
Ctenophora	*Mnemiopsis leidyi*	Genome Transcriptome	GCA_000226015.1 SRR1971277	Scaffold	–	–
Ctenophora	*Beroe ovata*	Genome	GCA_900239995.1	Contig	–	–
Placozoa	*Trichoplax adhaerens*	Genome	GCA_000150275.1	Scaffold	–	–
Placozoa	*Trichoplax*	Genome	GCA_003344405.1	Scaffold	–	–
Cnidaria	*Tripedalia cystophora*	Transcriptome	SRR8101523	n.a.	a	–
Cnidaria	*Nematostella vectensis*	Genome	GCA_000209225.1	Scaffold	XM_001634160.1	–
Cnidaria	*Stylophora pistillata*	Genome	GCA_002571385.1	Scaffold	XM_022924671.1	–
Cnidaria	*Pocillopora damicornis*	Genome	GCA_003704095.1	Scaffold	XM_027180944.1	–
Cnidaria	*Orbicella faveolata*	Genome	GCA_002042975.1	Scaffold	XM_020776224.1	–
Bilateria	*Drosophila melanogaster*	Genome	GCA_000001215.4	Chromosome	NM_166413.3	–
Bilateria	*Apis mellifera*	Genome	GCA_003254395.2	Chromosome	XM_001119966.5	–
Bilateria	*Caenorhabditis elegans*	Genome	GCA_000002985.3	Complete Genome	NM_059845.2	–
Bilateria	*Octopus bimaculoides*	Genome	GCA_001194135.1	Scaffold	XM_014922170.1	–
Bilateria	*Mizuhopecten yessoensis*	Genome	GCA_002113885.2	Scaffold	XM_021516578.1	–
Bilateria	*Meara stichopi*	Transcriptome	n.a.	n.a.	KY709787.1	–
Bilateria	*Acanthaster planci*	Genome	GCA_001949145.1	Scaffold	XM_022243979.1	–
Bilateria	*Saccoglossus kowalevskii*	Genome	GCA_000003605.1	Scaffold	NM_001164903.1	–
Bilateria	*Branchiostoma belcheri*	Genome	GCA_001625305.1	Scaffold	XM_019761392.1	–
Bilateria	*Ciona intestinalis*	Genome	GCA_000224145.2	Chromosome	NM_001032511.1	–
Bilateria	*Eptatretus burgeri*	Genome	GCA_900186335.2	Scaffold	ENSEBUT00000011203.1	–
Bilateria	*Petromyzon marinus*	Genome	GCA_002833325.1	Scaffold	b	–
Bilateria	*Callorhinchus milii*	Genome	GCA_000165045.2	Scaffold	XM_007903126.1	XM_007908006.1
Bilateria	*Erpetoichthys calabaricus*	Genome	GCA_900747795.2	Chromosome	XM_028803026.1	XM_028814691.1
Bilateria	*Acipenser ruthenus*	Genome	GCA_004119895.1	Scaffold	RXM93534.1	RXM94969.1
Bilateria	*Lepisosteus oculatus*	Genome	GCA_000242695.1	Chromosome	XM_006627139.2	XM_015365287.1
Bilateria	*Danio rerio*	Genome	GCA_000002035.4	Chromosome	NM_131227.1	NM_131225.2 NM_131226.2
Bilateria	*Takifugu rubripes*	Genome	GCA_901000725.2	Chromosome	XM_029837663.1	XM_003974173.2 XM_011616221.2
Bilateria	*Latimeria chalumnae*	Genome	GCA_000225785.1	Scaffold	XM_006005788.1	XM_005999053.1
Bilateria	*Xenopus tropicalis*	Genome	GCA_000004195.3	Chromosome	XM_002936669.4	XM_002941390.4
Bilateria	*Taeniopygia guttata*	Genome	GCA_003957565.2	Chromosome	NM_001243734.1	XM_030256513.1
Bilateria	*Monodelphis_domestica*	Genome	GCA_000002295.2	Chromosome	XM_007487510.2	XM_001373844.3
Bilateria	*Homo sapiens*	Genome	GCA_000001405.27	Chromosome	NM_013435.3	NM_032753.3
Bilateria	*Mus musculus*	Genome	GCA_000001635.8	Chromosome	NM_013833.2	–

^a^ The putative sequence of *Rax* ortholog was obtained from transcriptome

^b^ The putative sequence of lamprey *Rax* was directly retrieved from the genomic sequence using UCSC Genome Browser.

**Table 2:** Mammalian *Rax* and *Rax2* orthologs.

Group	Taxon name	Genome ID	Genome assembly level	*Rax*	*Rax2*
Euarchontoglires	*Homo sapiens*	GCA_000001405.27	Chromosome	NM_013435.3	NM_032753.3
Euarchontoglires	*Pan troglodytes*	GCA_002880755.3	Chromosome	XM_001142510.3	NM_001081487.1
Euarchontoglires	*Pan paniscus*	GCA_000258655.2	Chromosome	XM_008952703.1	XM_008972700.2
Euarchontoglires	*Pongo abelii*	GCA_002880775.3	Chromosome	XM_024236038.1	XM_009252600.2
Euarchontoglires	*Nomascus leucogenys*	GCA_000146795.3	Chromosome	XM_030810092.1	XM_004091072.2
Euarchontoglires	*Macaca mulatta*	GCA_000772875.3	Chromosome	XM_015122061.2	XM_002801027.3
Euarchontoglires	*Macaca fascicularis*	GCA_000364345.1	Chromosome	XM_005586557.2	XM_015440231.1
Euarchontoglires	*Macaca nemestrina*	GCA_000956065.1	Scaffold	XM_011733358.1	XM_011747810.2
Euarchontoglires	*Chlorocebus sabaeus*	GCA_000409795.2	Chromosome	XM_008013918.1	XM_007994799.1
Euarchontoglires	*Papio anubis*	GCA_000264685.2	Chromosome	XM_021930179.1	XM_003914667.1
Euarchontoglires	*Cercocebus atys*	GCA_000955945.1	Scaffold	XM_012069057.1	XM_012073070.1
Euarchontoglires	*Theropithecus gelada*	GCA_003255815.1	Chromosome	XM_025365606.1	XM_025368381.1
Euarchontoglires	*Mandrillus leucophaeus*	GCA_000951045.1	Scaffold	XM_011971742.1	XM_011967344.1
Euarchontoglires	*Piliocolobus tephrosceles*	GCA_002776525.2	Scaffold	XM_023218311.1	XM_023200859.2
Euarchontoglires	*Rhinopithecus bieti*	GCA_001698545.1	Scaffold	XM_017890457.1	XM_017847249.1
Euarchontoglires	*Rhinopithecus roxellana*	GCA_000769185.1	Scaffold	XM_030926113.1	XM_010367273.2
Euarchontoglires	*Colobus angolensis*	GCA_000951035.1	Scaffold	XM_011949591.1	XM_011943448.1
Euarchontoglires	*Callithrix jacchus*	GCA_000004665.1	Chromosome	XM_002757287.2	XM_002761582.3
Euarchontoglires	*Cebus capucinus*	GCA_001604975.1	Scaffold	XM_017528838.1	XM_017507399.1
Euarchontoglires	*Saimiri boliviensis boliviensis*	GCA_000235385.1	Scaffold	XM_010336873.1	XM_010349647.1
Euarchontoglires	*Aotus nancymaae*	GCA_000952055.2	Scaffold	XM_012446398.1	XM_012436148.2
Euarchontoglires	*Otolemur garnettii*	GCA_000181295.3	Scaffold	XM_003788425.1	XM_003788870.2
Euarchontoglires	*Propithecus coquereli*	GCA_000956105.1	Scaffold	XM_012651751.1	XM_012646140.1
Euarchontoglires	*Microcebus murinus*	GCA_000165445.3	Chromosome	XM_012745461.1	XM_012769405.1
Euarchontoglires	*Galeopterus variegatus*	GCA_000696425.1	Scaffold	XM_008580185.1	XM_008582889.1
Euarchontoglires	*Tupaia chinensis*	GCA_000334495.1	Scaffold	XM_006139947.2	XM_006171790.2
Euarchontoglires	*Ochotona princeps*	GCA_000292845.1	Scaffold	XM_004579462.1	–
Euarchontoglires	*Ictidomys tridecemlineatus*	GCA_000236235.1	Scaffold	XM_013357567.2	XM_021730889.1
Euarchontoglires	*Urocitellus parryii*	GCA_003426925.1	Scaffold	XM_026393165.1	XM_026404447.1
Euarchontoglires	*Marmota flaviventris*	GCA_003676075.1	Scaffold	XM_027948970.1	XM_027952705.1
Euarchontoglires	*Mus musculus*	GCA_000001635.8	Chromosome	NM_013833.2	–
Euarchontoglires	*Rattus norvegicus*	GCA_000001895.4	Chromosome	NM_053678.1	–
Euarchontoglires	*Nannospalax galili*	GCA_000622305.1	Scaffold	XM_008840033.1	–
Euarchontoglires	*Cavia porcellus*	GCA_000151735.1	Scaffold	XM_013157082.1	–
Euarchontoglires	*Octodon degus*	GCA_000260255.1	Scaffold	XM_004647907.1	–
Euarchontoglires	*Dipodomys ordii*	GCA_000151885.2	Scaffold	XM_013018940.1	–
Laurasiatheria	*Panthera pardus*	GCA_001857705.1	Scaffold	XM_019463885.1	XM_019430847.1
Laurasiatheria	*Felis catus*	GCA_000181335.4	Chromosome	XM_023242049.1	XM_023243834.1
Laurasiatheria	*Canis lupus familiaris*	GCA_000002285.2	Chromosome	XM_022423505.1	XM_849723.4
Laurasiatheria	*Vulpes vulpes*	GCA_003160815.1	Scaffold	XM_025997549.1	XM_026019439.1
Laurasiatheria	*Ailuropoda melanoleuca*	GCA_000004335.1	Scaffold	XM_011231633.1	XM_002923561.3
Laurasiatheria	*Ursus arctos horribilis*	GCA_003584765.1	Scaffold	XM_026495947.1	XM_026481074.1
Laurasiatheria	*Ursus maritimus*	GCA_000687225.1	Scaffold	XM_008705444.1	XM_008711252.1
Laurasiatheria	*Leptonychotes weddellii*	GCA_000349705.1	Scaffold	XM_006734844.1	XM_006750976.2
Laurasiatheria	*Neomonachus schauinslandi*	GCA_002201575.1	Scaffold	XM_021686390.1	XM_021705428.1
Laurasiatheria	*Eumetopias jubatus*	GCA_004028035.1	Scaffold	XM_028102872.1	XM_028126063.1
Laurasiatheria	*Zalophus californianus*	GCA_900631625.1	Scaffold	XM_027576397.1	XM_027587801.1
Laurasiatheria	*Mustela putorius furo*	GCA_000215625.1	Scaffold	XM_004745687.2	–
Laurasiatheria	*Manis javanica*	GCA_001685135.1	Scaffold	XM_017662163.1	XM_017641956.1
Laurasiatheria	*Equus caballus*	GCA_002863925.1	Scaffold	XM_023647900.1	XM_023644413.1
Laurasiatheria	*Equus asinus*	GCA_001305755.1	Scaffold	XM_014859029.1	XM_014843046.1
Laurasiatheria	*Ceratotherium simum simum*	GCA_000283155.1	Scaffold	XM_004422590.2	XM_004441310.2
Laurasiatheria	*Lagenorhynchus obliquidens*	GCA_003676395.1	Scaffold	XM_027118662.1	XM_027086050.1
Laurasiatheria	*Orcinus orca*	GCA_000331955.2	Scaffold	XM_004268055.1	XM_004277200.1
Laurasiatheria	*Lipotes vexillifer*	GCA_000442215.1	Scaffold	XM_007450117.1	XM_007460506.1
Laurasiatheria	*Neophocaena asiaeorientalis asiaeorientalis*	GCA_003031525.1	Scaffold	XM_024754865.1	XM_024745933.1
Laurasiatheria	*Delphinapterus leucas*	GCA_002288925.2	Scaffold	XM_022584361.2	XM_022557349.2
Laurasiatheria	*Physeter catodon*	GCA_002837175.1	Scaffold	XM_024133906.1	XM_007109282.2
Laurasiatheria	*Balaenoptera acutorostrata*	GCA_000493695.1	Scaffold	XM_007192060.1	XM_007169190.1
Laurasiatheria	*Ovis aries*	GCA_002742125.1	Chromosome	XM_027960933.1	XM_027969870.1
Laurasiatheria	*Capra hircus*	GCA_001704415.1	Chromosome	XM_018039348.1	XM_005682656.3
Laurasiatheria	*Bubalus bubalis*	GCA_003121395.1	Chromosome	XM_025273363.1	XM_006047896.2
Laurasiatheria	*Bison bison bison*	GCA_000754665.1	Scaffold	XM_010854825.1	XM_010828446.1
Laurasiatheria	*Bos taurus*	GCA_002263795.2	Chromosome	XM_024984497.1	NM_182653.1
Laurasiatheria	*Bos mutus*	GCA_000298355.1	Scaffold	XM_005911824.1	XM_005895951.1
Laurasiatheria	*Bos indicus*	GCA_000247795.2	Chromosome	XM_019986442.1	XM_019963601.1
Laurasiatheria	*Sus scrofa*	GCA_000003025.6	Chromosome	XM_003121712.3	XM_005661348.3
Laurasiatheria	*Camelus dromedarius*	GCA_000767585.1	Scaffold	XM_031441660.1	XM_010996050.2
Laurasiatheria	*Camelus bactrianus*	GCA_000767855.1	Scaffold	XM_010961429.1	XM_010966706.1
Laurasiatheria	*Vicugna pacos*	GCA_000164845.3	Scaffold	XM_006205802.1	XM_006206395.1
Laurasiatheria	*Miniopterus natalensis*	GCA_001595765.1	Scaffold	XM_016223487.1	XM_016197969.1
Laurasiatheria	*Hipposideros armiger*	GCA_001890085.1	Scaffold	XM_019640612.1	XM_019653323.1
Laurasiatheria	*Desmodus rotundus*	GCA_002940915.2	Scaffold	XM_024580521.1	XM_024569247.1
Laurasiatheria	*Rhinolophus sinicus*	GCA_001888835.1	Scaffold	XM_019729367.1	XM_019744054.1
Laurasiatheria	*Pteropus alecto*	GCA_000325575.1	Scaffold	XM_025048123.1	XM_006904102.2
Laurasiatheria	*Pteropus vampyrus*	GCA_000151845.2	Scaffold	XM_011361137.2	XM_011372678.2
Laurasiatheria	*Erinaceus europaeus*	GCA_000296755.1	Scaffold	XM_007529018.1	–
Laurasiatheria	*Sorex araneus*	GCA_000181275.2	Scaffold	XM_004601992.1	–
Laurasiatheria	*Condylura cristata*	GCA_000260355.1	Scaffold	XM_004684020.1	XM_004688892.1
Afrotheria	*Loxodonta africana*	GCA_000001905.1	Scaffold	XM_003406278.1	–
Afrotheria	*Trichechus manatus latirostris*	GCA_000243295.1	Scaffold	XM_023733937.1	XM_004378466.1
Afrotheria	*Chrysochloris asiatica*	GCA_000296735.1	Scaffold	XM_006837574.1	XM_006869042.1
Afrotheria	*Echinops telfairi*	GCA_000313985.1	Scaffold	XM_004703116.1	XM_004714381.1
Afrotheria	*Elephantulus edwardii*	GCA_000299155.1	Scaffold	XM_006892750.1	XM_006897892.1
Afrotheria	*Orycteropus afer*	GCA_000298275.1	Scaffold	XM_007935546.1	XM_007951029.1
Xenarthra	*Dasypus novemcinctus*	GCA_000208655.2	Scaffold	XM_004447204.1	XM_004447500.3

### Multiple sequence alignment of the octapeptide, homeodomain, or OAR motif in *Rax* orthologs

Amino acid sequences of the octapeptide, homeodomain, or OAR motif in *Rax* orthologs were aligned by clustal omega with the default parameters [28]. Resulting multiple sequence alignments were verified and visualized by jalview [29].

### Molecular phylogenetic analysis of Rax and Rax2 of jawed vertebrates and Rax of jawless vertebrates

Amino acid sequences of Rax and Rax2 of jawed vertebrates and Rax of jawless vertebrates were aligned using clustal omega [28] and muscle [30] under default parameters. The multiple sequence alignment results were verified and visualized using jalview [29]. Maximum‐likelihood trees were constructed using the Poisson, Whelan and Goldman (WAG), Le–Gascuel (LG), or Jones–Taylor–Thornton (JTT) models using mega7 [31]. Neighbor‐joining trees were constructed with the Poisson, Dayhoff, or JTT models using mega7 [31]. All positions with < 90% site coverage were excluded from analyses. In other words, fewer than 10% alignment gaps or missing data were allowed at any position. The bootstrap values were estimated from 500 replicates in all analyses.

### Synteny analysis of mammalian *Rax* and *Rax2*

Genome sequences and annotations were obtained from the NCBI Assembly database. In this database, the genome assembly quality is classified into four categories, in order of highest to lowest quality: complete genome, chromosome, scaffold, and contig (https://www.ncbi.nlm.nih.gov/assembly/help/). We used genome assemblies of scaffold to complete genome quality in this analysis. The arrangements of genes around *Rax* or *Rax2* were compared between species. Even if a relatively high‐quality genome assembly is used for analysis, low‐quality regions are often included to some extent due to low sequencing read coverage or the presence of repetitive sequences [32]. Therefore, regions with fewer than three genes surrounding *Rax* or *Rax2* were excluded. We reported the genome analysis results where *Rax* and *Rax2* loci were identified.

### Maximum‐likelihood tree construction based on mammalian Rax and Rax2 alignment

The amino acid sequences of 86 placental mammalian Rax, 75 placental mammalian Rax2, opossum Rax, and opossum Rax2 protein sequences were aligned using clustal omega under default parameters [28]. Based on this alignment, a maximum‐likelihood tree was constructed with the JTT model using MEGA7 [31]. All positions with < 90% site coverage were excluded from analyses. Bootstrap values were estimated from 500 replicates. The complete list of accession numbers for the analyzed protein sequences is provided in Table 2.

### Calculation of nonsynonymous‐to‐synonymous substitution ratio

The amino acid sequences of 86 placental mammalian Rax, 75 placental mammalian Rax2, opossum Rax, and opossum Rax2 protein sequences were aligned using clustal omega under default parameters as described above [28]. Based on this alignment and the set of protein‐coding sequences, we generated codon alignment using tranalign [33]. By comparing human *RAX* and *RAX2* with respective mammalian *Rax* and *Rax2* orthologs, we calculated nonsynonymous‐to‐synonymous rate ratios (Ka/Ks) for each ortholog pair using the Jukes–Cantor model. All positions with < 90% site coverage in the alignment were excluded from analyses. The average *Rax* Ka/Ks and *Rax2* Ka/Ks were compared using a Welch two‐sample *t*‐test. The difference was considered statistically significant if the *P* value was < 0.05.

## Results

### Identification of *Rax* in various animal species

**Fig. 1:**
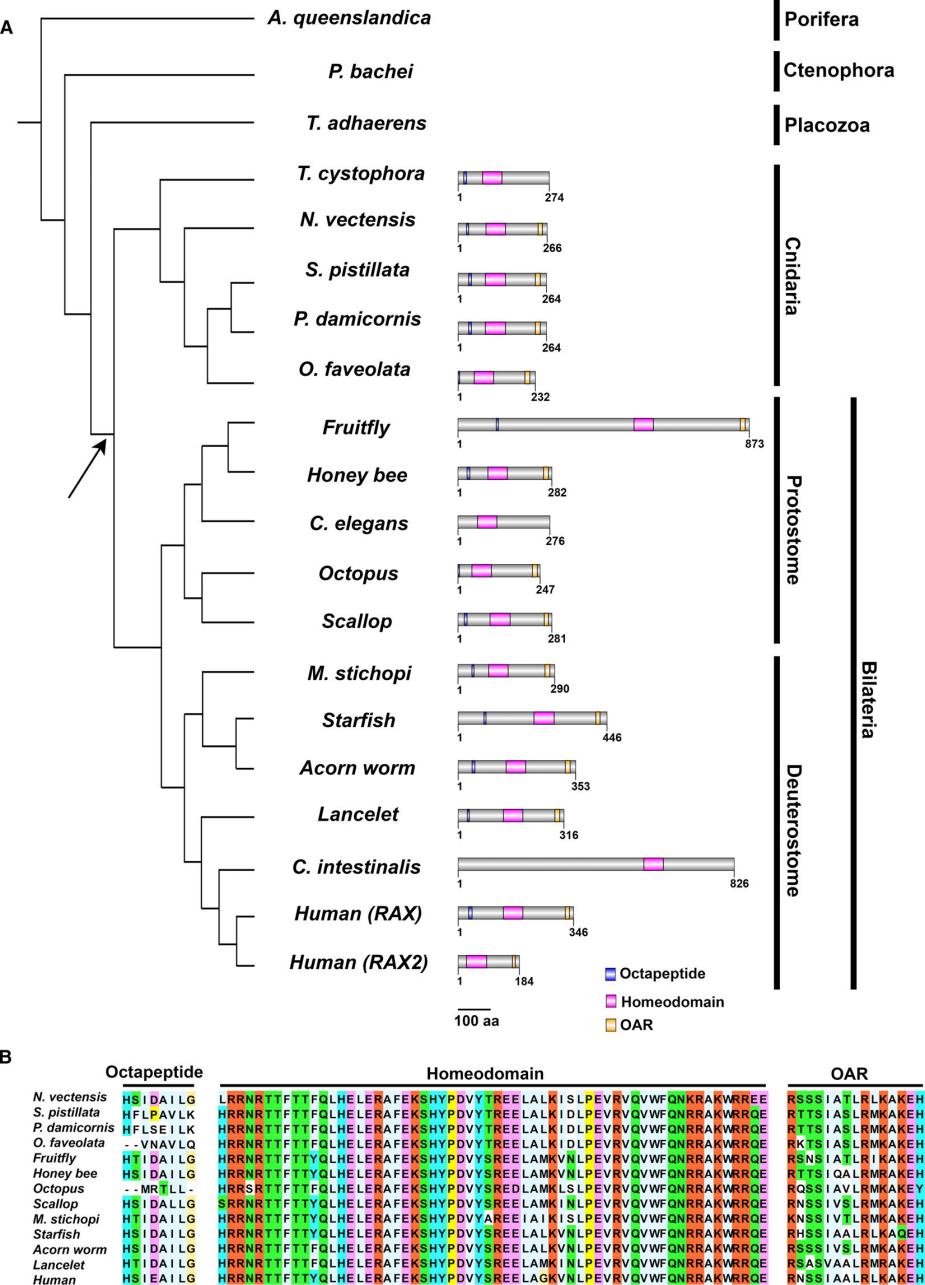
Phylogeny of animal species and their *Rax* genes. (A) Cladogram of representative animals and domain organizations of their *Rax* genes. Note the absence of *Rax* in Ctenophora, Porifera, and Placozoa. Blue boxes indicate octapeptides, magenta boxes indicate homeodomains, and yellow boxes indicate OAR motifs. The cladogram topology is derived from previous studies [54, 55, 56, 57]. It should be noted that the Metazoan phylogeny is controversial. The arrow indicates the presumed origin of the *Rax* gene. The scale bar indicates 100 amino acid residues. (B) Sequence alignments of the octapeptide, homeodomain, and OAR motif in *Rax* orthologs. These three domains/motifs are well conserved in Cnidaria and Bilateria. Each residue is colored according to the Clustal X residue code [58].

To investigate the evolutionary origin of *Rax*, we comprehensively searched for *Rax* in the genomes or transcriptomes of animals that are evolutionarily distant from each other. The criteria to determine whether a *Rax* ortholog is present in a species are described in Materials and Methods. This analysis included genome sequences of 34 animal species: two Porifera, three Ctenophora, two Placozoa, four Cnidaria, and 23 Bilateria (Fig. 1A, Table 1). Transcriptome data from *Sycon ciliatum*, a Porifera organism, and *Mnemiopsis leidyi*, a Ctenophora organism, were also analyzed. In all Cnidaria and Bilateria analyzed, we identified *Rax* orthologs (Fig. 1A). In contrast, we did not identify any *Rax* gene in Porifera, Ctenophore, and Placozoa, which are groups phylogenetically more distant from both Bilateria and Cnidaria (Fig. 1A). Most of the *Rax* genes in Cnidaria and Bilateria were shown to have all of the octapeptide, homeodomain, and OAR motif (Fig. 1A). Sequence alignment analyses revealed that amino acid sequences of octapeptide, homeodomain, and OAR motif are highly conserved among these animals (Fig. 1B). These results suggest that *Rax* appeared in the common ancestor of Bilateria and Cnidaria, and has been highly conserved in terms of domain organization and sequence similarity over the course of evolution.

### Phylogenetic analysis of *Rax* and *Rax2* in jawed and jawless vertebrates

**Fig. 2:**
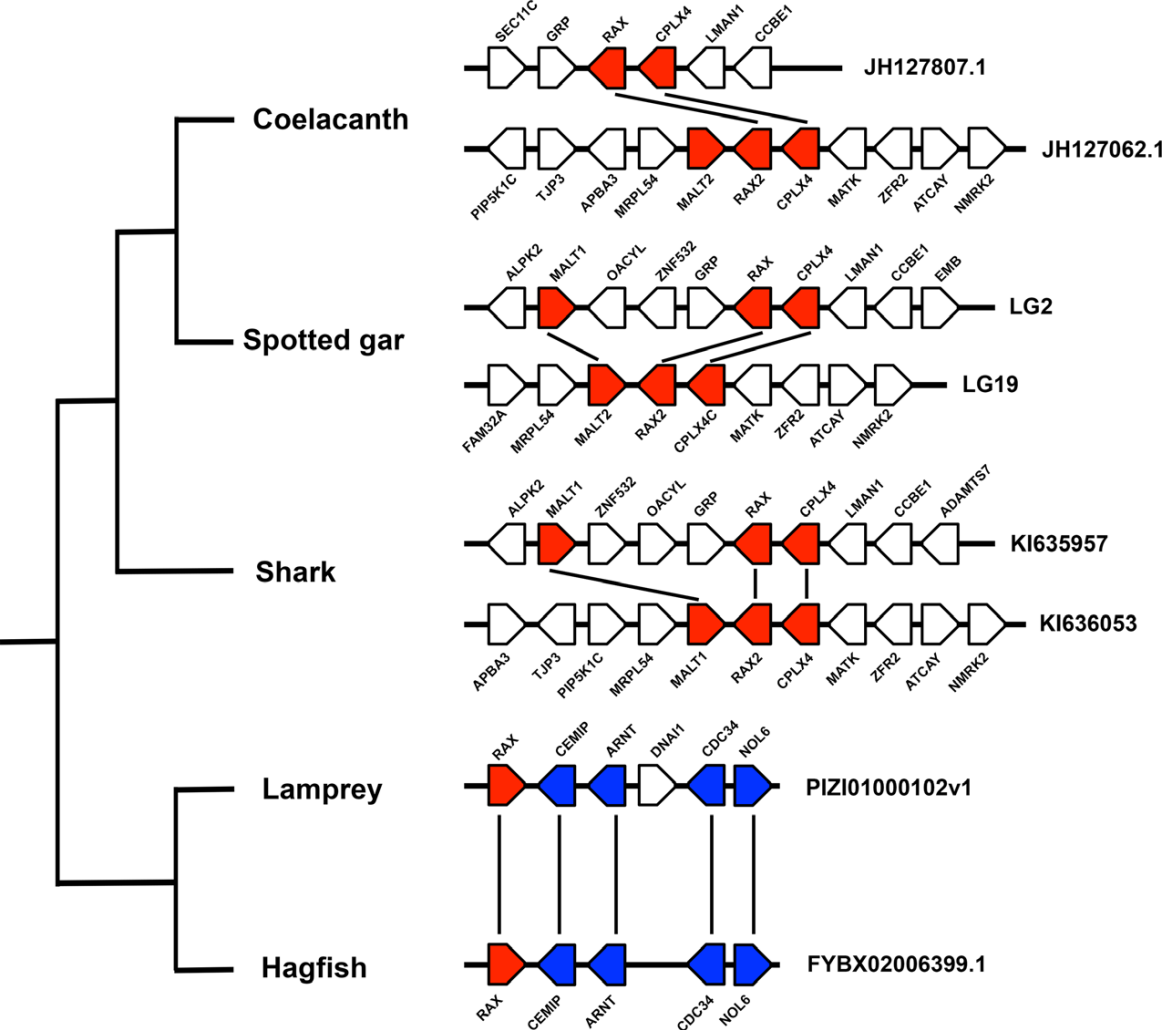
Evolution of *Rax* and *Rax2* gene structures in vertebrates. Synteny around *Rax* and *Rax2* genes in basal vertebrates. While jawed vertebrates possess both *Rax* and *Rax2*, jawless vertebrates possess a single *Rax*. Black lines indicate paralogous or orthologous relationships. Scaffold names are indicated on the right of the panel.

Vertebrates are divided into two major groups, jawed and jawless, depending on whether the jaw is present. Jawed vertebrates possess *Rax2* in addition to *Rax* [12, 21]. The high sequence similarity of jawed vertebrate Rax and Rax2 suggests that they resulted from gene duplication. We analyzed the genomes of jawed and jawless vertebrate species to estimate the evolutionary timepoint when these two genes appeared. We analyzed the genomes of elephant shark, spotted gar, and coelacanth as representatives of jawed vertebrates. On the other hand, as representative of jawless vertebrates, we analyzed the genomes of lamprey and hagfish, whose genomes were recently sequenced. In jawed vertebrates, we identified both *Rax* and *Rax2* genes (Fig. 2). The arrangement of the genes *Malt1‐Rax‐Cplx4* and their paralogous counterparts was conserved in these organisms. In contrast, we identified only single *Rax* in both lamprey and hagfish (Fig. 2). Notably, the arrangements of genes around *Rax* in lamprey or hagfish were very similar, suggesting that their *Rax* genes are orthologous to each other (Fig. 2).

**Fig. 3:**
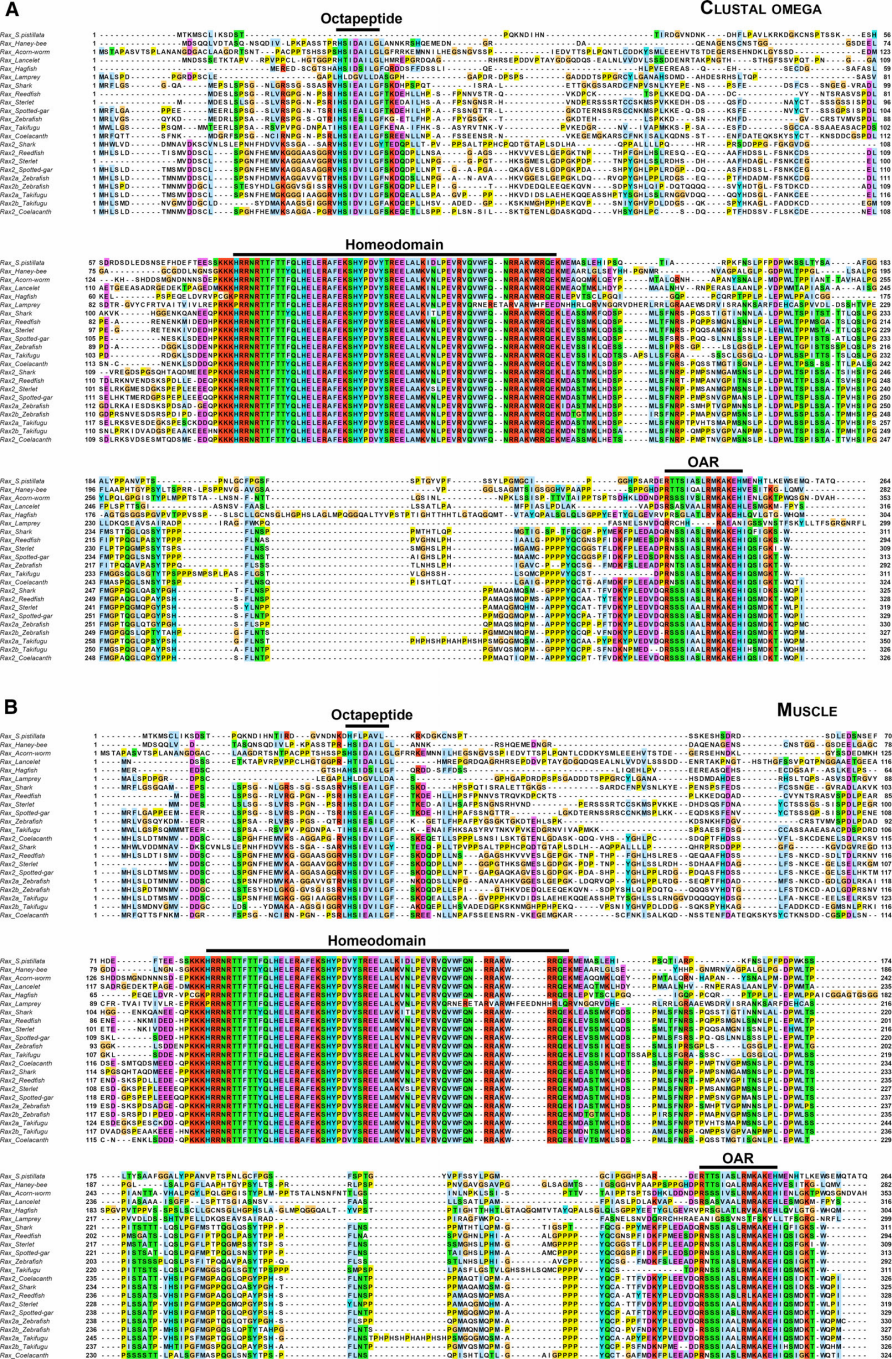
Multiple sequence alignments of Rax orthologs. (A) Multiple sequence alignment of Rax orthologs using clustal omega. The 22 protein sequences of Rax were aligned by clustal omega [28]. (B) Multiple sequence alignment of Rax orthologs using muscle. The same set of protein sequences in (A) were aligned by muscle [30]. Each residue is colored according to the Clustal X residue code [58].

**Fig. 4:**
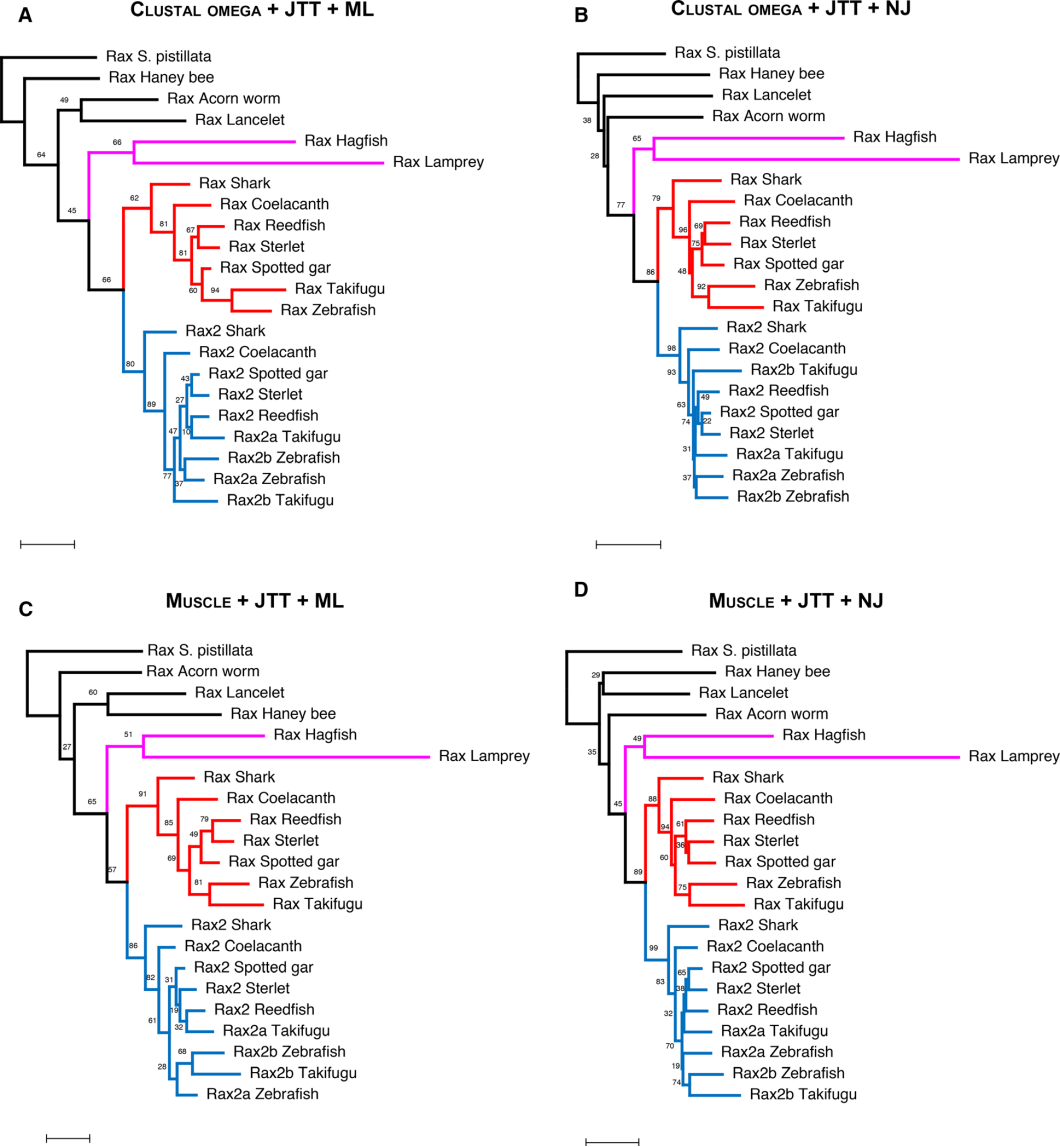
Molecular phylogenetic analysis of Rax and Rax2 in various animal species. Multiple sequence alignments were used to construct maximum‐likelihood trees from (A) clustal omega or (C) muscle and neighbor‐joining trees from (B) clustal omega or (D) muscle. In all these analyses, the JTT model was used as the amino acid substitution model. Jawed vertebrate Rax sequences are colored in red. Jawed vertebrate Rax2 sequences are colored in blue. Lamprey and hagfish Rax sequences are colored in magenta. The scale bars represent 0.2 amino acid substitutions per site. Bootstrap values are given on each node.

We next analyzed the phylogenetic relationships between jawless vertebrate Rax, jawed vertebrate Rax, and jawed vertebrate Rax2. Tetrapod Rax2 lacks the N‐terminal region, including octapeptide, and is shorter than Rax [21]. Therefore, we focused on nontetrapod vertebrate Rax2 for molecular phylogenetic analysis in order to obtain as much phylogenetic information from the sequence alignments as possible. A total of 22 protein sequences were analyzed, including seven jawed vertebrate Rax, nine jawed vertebrate Rax2, two jawless vertebrate Rax, and four invertebrate Rax. To perform robust molecular phylogenetic analysis, we used the clustal omega [28] and muscle [30] programs to generate two multiple protein sequence alignments (Fig. 3). Based on these alignments, we constructed phylogenetic trees using the maximum‐likelihood method in the character state methods and the neighbor‐joining trees in the distance matrix methods (Fig. 4, Figs S1 and S2). Maximum‐likelihood trees were constructed using the Poisson, WAG, LG, or JTT models. Neighbor‐joining trees were constructed using the Poisson, Dayhoff, or JTT models. In all cases, lamprey and hagfish *Rax* formed a sister group to *Rax* and *Rax2 *of jawed vertebrates (Fig. 4, Figs S1 and S2). Taken together, the current synteny analysis and molecular phylogenetic analysis suggest that *Rax* and *Rax2* of jawed vertebrates resulted from segmental duplication of a small region containing *Malt1*, *Rax*, and *Cplx4* ancestors that occurred after jawed vertebrates diverged from jawless ones.

### Comparative analysis of *Rax* and *Rax2* gene structures in vertebrates

**Fig. 5:**
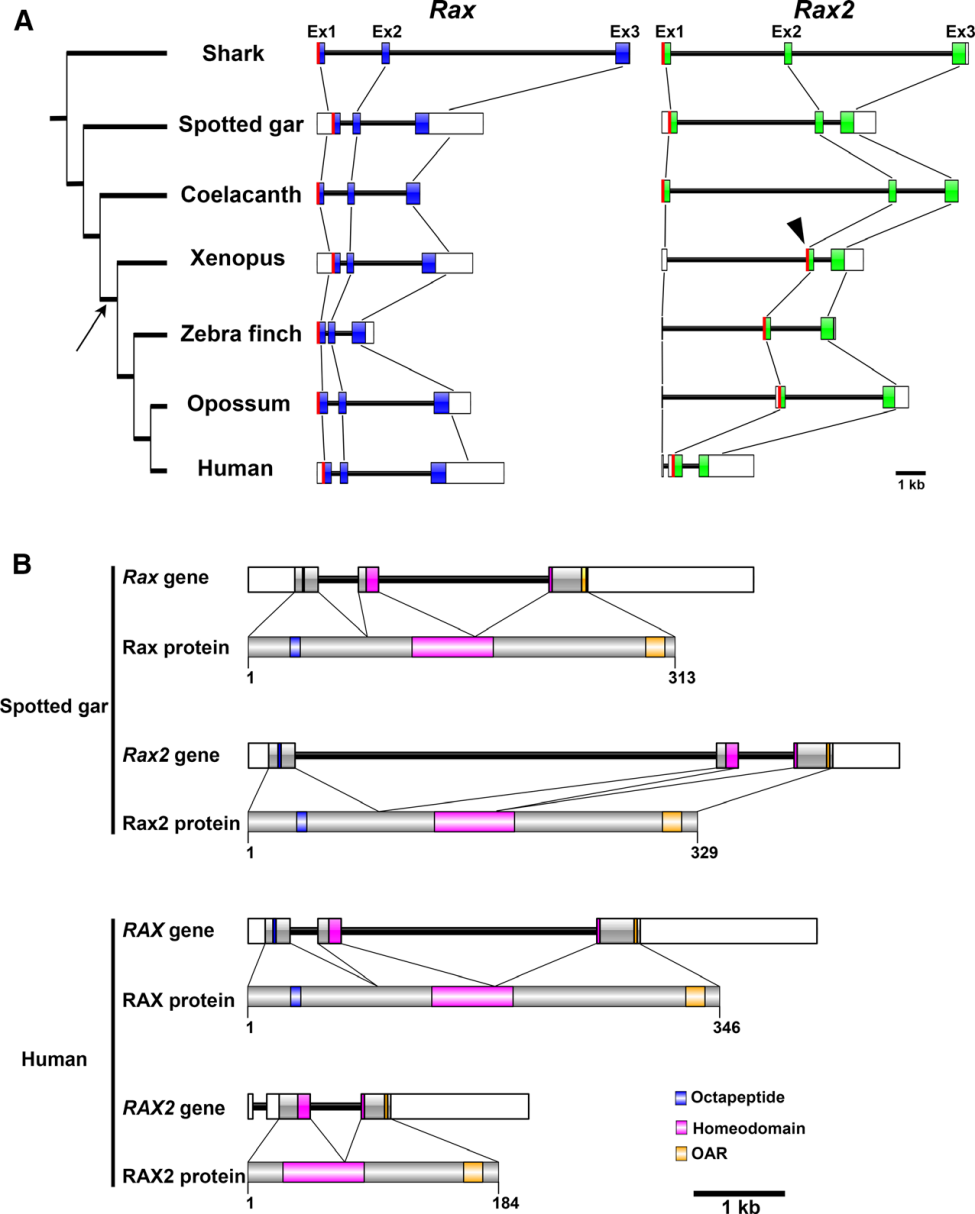
*Rax* and *Rax2* gene structures. (A) *Rax* and *Rax2* gene structures of seven vertebrates. All *Rax* and *Rax2* genes are composed of three exons. Protein‐coding sequences of *Rax* or *Rax2* are colored blue or green, respectively. In shark, spotted gar, and coelacanth, start codons of *Rax2* (red line) are located on the first exon. In Xenopus, zebra finch, opossum, and human, start codons of *Rax2* are shifted to the second exon (arrowhead). The arrow indicates the presumed point of *Rax2* octapeptide loss. The scale bar indicates 1 kbp. (B) *Rax* and *Rax2* gene structures of spotted gar and human. *Rax* and *Rax2* gene structures and their respective protein domain/motif architectures are shown. Note that human *RAX2* has its start codon at the second exon and lacks the octapeptide. Blue boxes indicate octapeptides, magenta boxes indicate homeodomains, and yellow boxes indicate OAR motifs. The scale bar indicates 1 kbp.

Tetrapod *Rax2* genes were reported to lack octapeptide domains [21]. To investigate how the loss of octapeptide in tetrapods occurred, we compared the gene structures of vertebrate *Rax* and *Rax2*. We included seven vertebrates from shark to human in this analysis (Fig. 5A). In all species analyzed, *Rax* or *Rax2* gene was composed of three exons (Fig. 5A). In both genes, octapeptides were coded in the first exon (Fig. 5B). Start codons of *Rax* were located on the first exons in all species (Fig. 5A). Similarly, start codons of *Rax2* in shark, spotted gar, and coelacanth were located on the first exon; however, in all tetrapods analyzed, start codons of *Rax2* were shifted to the second exon, resulting in the loss of octapeptides (Fig. 5A).

### Identification of *Rax2* gene loss events in mammals

**Fig. 6:**
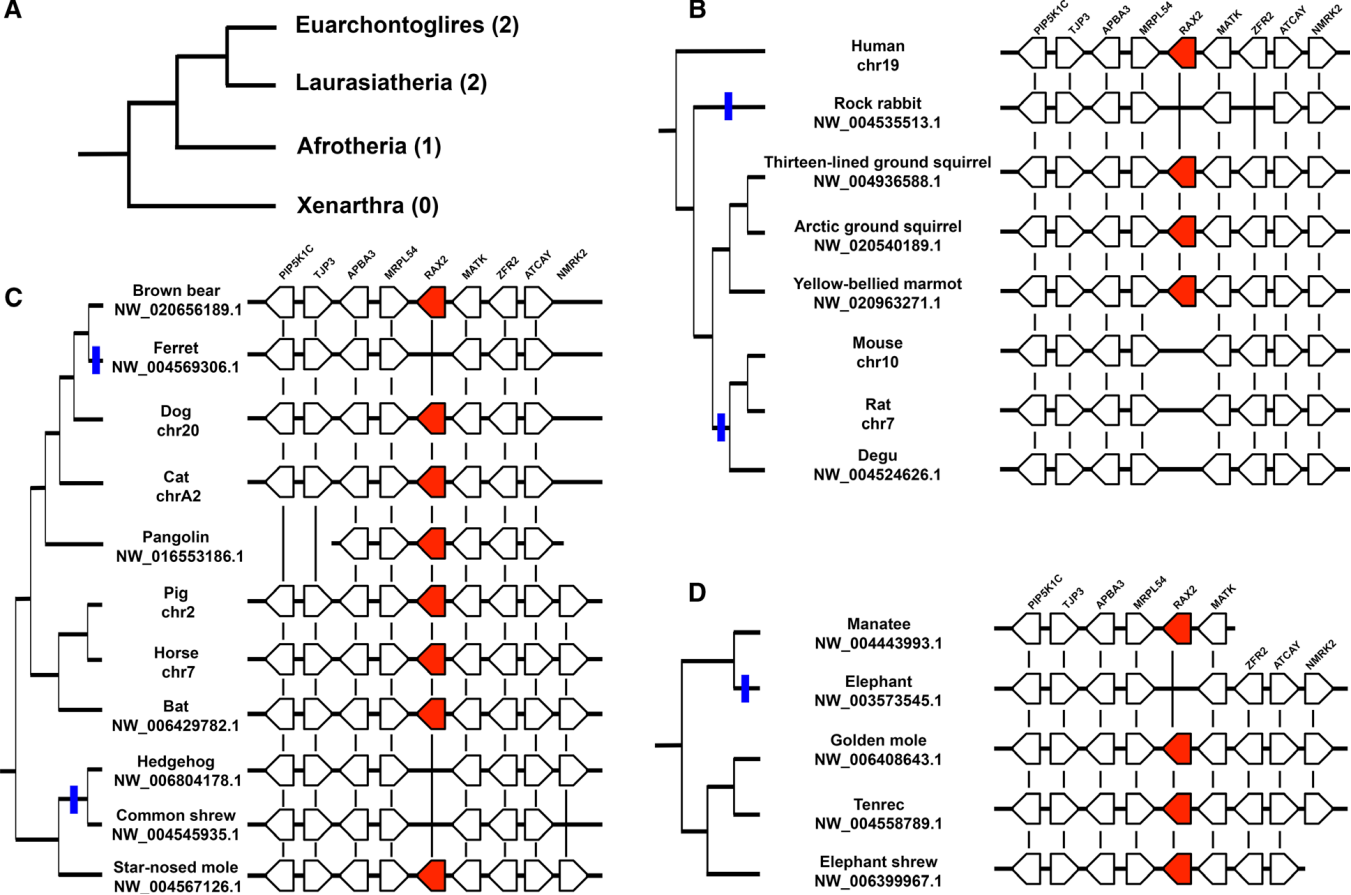
*Rax2* gene losses in mammals. (A) Phylogeny of mammals and *Rax2* gene loss. Mammals are divided into four major groups. Numbers of *Rax2* gene loss events are given in parentheses. The topology of this cladogram is based on previous reports [59, 60, 61]. (B) Synteny of the *Rax2* locus in Euarchontoglires. Rock rabbit and a subgroup of rodents, including mouse, rat, and degu, show independent *Rax2* gene loss (blue lines). (C) Synteny of *Rax2* locus in Laurasiatheria. There are two independent *Rax2* gene loss events in Laurasiatheria (blue lines). (D) Synteny of *Rax2* locus in Afrotheria. Elephant lacks the *Rax2* gene (blue line). *Rax2* genes are colored red. Black lines indicate orthologous relationships. Scaffold name is shown below each taxon name.

Since mice are known to lack the *Rax2* gene [12], we investigated whether more *Rax2* gene loss events have occurred in mammals by comparative analysis of *Rax* and *Rax2* loci in mammals. Mammals are phylogenetically divided into four major groups: Euarchontoglires, Laurasiatheria, Afrotheria, and Xenarthra (Fig. 6A). We examined both *Rax* and *Rax2* loci in 86 mammalian genomes (Table 2). While all 86 *Rax* loci contained *Rax* genes, 11 *Rax2* loci lacked the *Rax2* gene (Table 2). Of the investigated Euarchontoglires, rock rabbit and six rodent species lack *Rax2* (Fig. 6B, Table 2). Notably, three squirrel species possess *Rax2* genes, suggesting that an ancestor of rodents and one of the rabbits independently lost *Rax2* genes (Fig. 6B, Table 2). In Laurasiatheria, ferret, hedgehog, and common shrew lack *Rax2* genes (Fig. 6C, Table 2). Recent studies on the Eulipotyphla phylogeny indicated that hedgehog and common shrew are the sister group to star‐nosed mole [34, 35]. Since star‐nosed mole has the *Rax2* gene, a single *Rax2* gene loss event appears to have occurred in the common ancestor of hedgehog and common shrew. In Afrotheria, elephant lacks the *Rax2* gene (Fig. 6D, Table 2). In Xenarthra, we analyzed the armadillo genome and identified the *Rax2* gene. In summary, we detected five independent gene loss events of the *Rax2* gene in mammals (Fig. 6A).

### Molecular phylogenetic analysis of mammalian Rax and Rax2

**Fig. 7:**
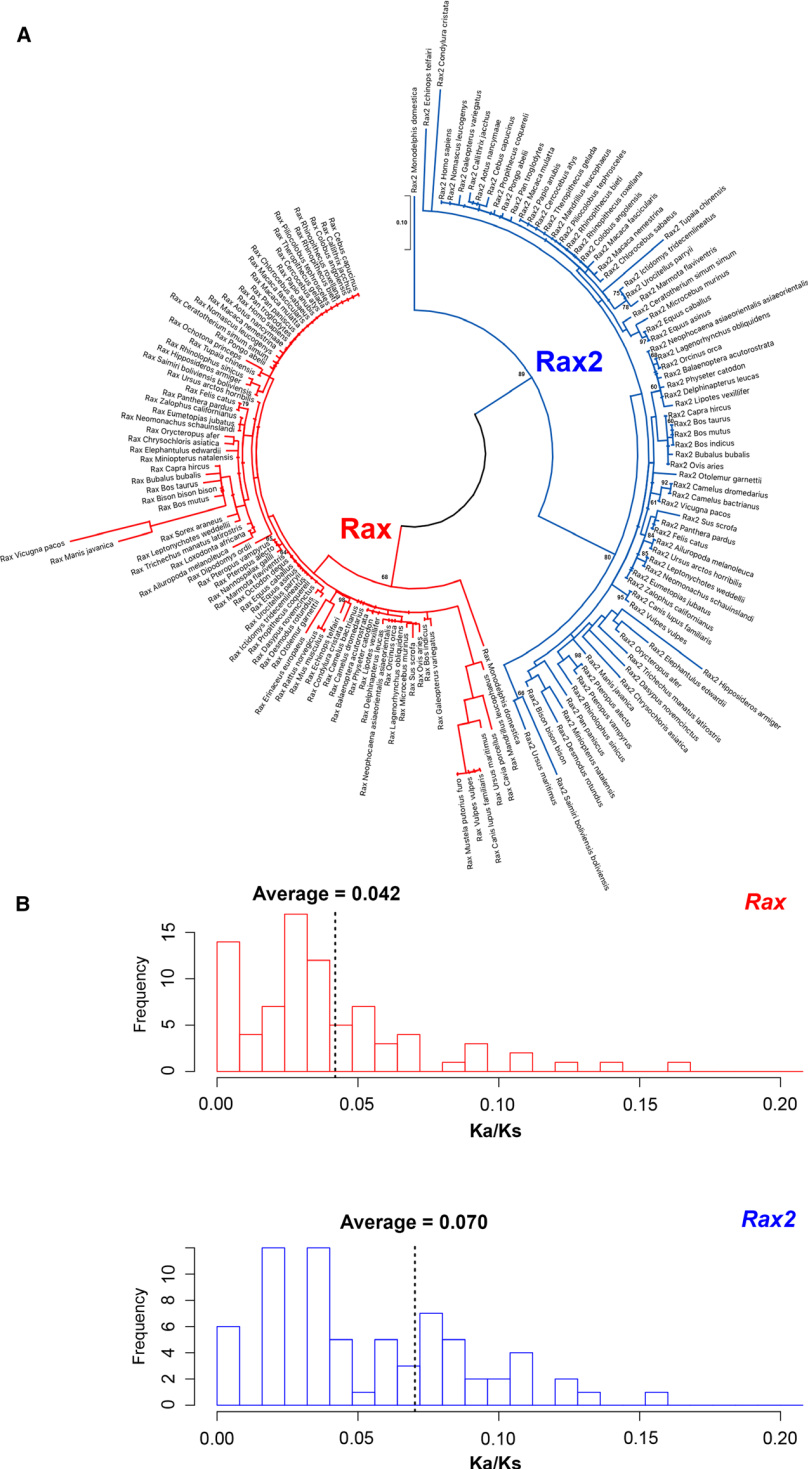
Phylogenetic analysis of mammalian Rax and Rax2. (A) A maximum‐likelihood tree of mammalian Rax and Rax2. A maximum‐likelihood tree was constructed from the amino acid sequence alignment containing Rax (red) and Rax2 (blue) from 86 placental mammals and opossum (*Monodelphis domestica*), a marsupial. The scale bars represent 0.1 amino acid substitutions per site. Bootstrap values > 0.6 are given on each node. (B) Ka/Ks ratio distributions of mammalian *Rax* (upper panel) or *Rax2* (lower panel). Ka/Ks ratios comparing human *RAX* and *RAX2* with respective mammalian *Rax* and *Rax2* were calculated. The average Ka/Ks values are indicated by dotted lines.

In order to further analyze mammalian Rax and Rax2 evolution, we aligned all 86 Rax and 75 Rax2 protein sequences identified in the current study and constructed a maximum‐likelihood tree (Fig. 7A, Table 2). As expected, Rax and Rax2 formed a monophyletic group in the tree (Fig. 7A). The Rax2 branch lengths appeared to be longer than the Rax lengths. Therefore, to quantitatively compare the degree of amino acid substitutions, we compared the nucleotide substitutions between human *RAX* and *RAX2* with their respective mammalian *Rax* and *Rax2* orthologs. Then, we calculated Ka/Ks ratios for each ortholog gene pair. The *Rax2* Ka/Ks values showed a broader distribution compared to *Rax* (Fig. 7B). The average Ka/Ks value was 0.042 for *Rax* and 0.070 for *Rax2* (Fig. 7B), indicating that the average mammalian *Rax2* Ka/Ks value was ~ 67% greater than mammalian Rax (*P* < 0.01, Welch two‐sample *t*‐test; Fig. 7B).

## Discussion

Our comprehensive analysis of *Rax* orthologs suggests that *Rax* appeared after Bilateria and Cnidaria diverged from other lineages over the course of evolution (Fig. 1A). It has remained unknown whether *Rax* is present in species evolutionarily distant from Bilateria and Cnidaria, probably because genome sequences of such distant species remained unavailable [23]. Fortunately, genome sequences of such distant species have recently become available, including Porifera, Ctenophora, and Placozoa, providing us with a valuable resource for comparative genomics [20]. Consistent with the previous studies, we definitively showed that Cnidaria and Bilateria possess *Rax*, whereas Placozoa does not (Fig. 1A) [22, 23]. Conversely, we showed that Porifera and Ctenophora may lack *Rax* using a comprehensive analysis of their genomes or transcriptomes (Fig. 1A, Table 1). It has been proposed that the *Hox* genes diversified due to rapid gene duplication before diversification of Cnidaria and Bilateria [36]. Similarly, ancestral paired‐type homeobox genes may have diverged, resulting in the appearance of the *Rax* gene before the diversification of Cnidaria and Bilateria. However, the divergence between Placozoa and the common ancestor of Cnidaria and Bilateria is very ancient. Therefore, our analysis cannot exclude the possibility that highly accumulated substitutions affect our *Rax* ortholog search results. The incompleteness of the genome assemblies should also be considered because all genome assemblies for Porifera, Ctenophora, and Placozoa are assembled at the contig or scaffold level. We also showed that the domain organization and amino acid sequences of the octapeptide, homeodomain, and OAR motif are highly conserved between cnidarian *Rax* and bilaterian *Rax* (Fig. 1B). Based on these observations, we propose that the origin of the *Rax* gene dates back to the common ancestor of Cnidaria and Bilateria and that *Rax* is highly conserved among Cnidaria and Bilateria.

From the very simple structure seen in Porifera to more complex structures, animal body plans have become more elaborate over the course of evolution [37]. Cnidaria were the first animal organisms to develop nervous systems [38]. Moreover, some Cnidaria in the medusozoan group display complex lens‐containing eyes [39, 40]. Since the current analysis suggests that *Rax* appeared in the common ancestor of Cnidaria and Bilateria, the evolutionary appearance of *Rax* might underlie the evolution of the eye in this ancestor. Functional analysis of *Rax* in extant Cnidaria may provide important clues to clarify the evolution of the eye.

*Pax6* is a paired‐type homeobox gene [13], which is an ortholog of *eyeless* in flies and plays a critical role in eye formation in both flies and mammals. However, its evolutionary origin is after the divergence of Bilateria and Cnidaria [41, 42]. Although the cnidarian *PaxB* gene is considered to be related to *Pax6* in Bilateria, the domain organization and DNA‐binding specificity of the paired domain differ between these two genes [41, 42]. In contrast, cnidarian *Rax* has the same domain organization as bilaterian *Rax*. Furthermore, amino acid sequences of the octapeptide, homeodomain, and OAR motif are highly conserved between cnidarian *Rax* and bilaterian *Rax* (Fig. 1B). Based on these observations, we propose the following scenario for the roles of Rax and Pax6 in eye evolution: *Rax* appeared in the common ancestor of Bilateria and Cnidaria, predating *Pax6* in terms of the evolutionary origin and the involvement in eye formation; after the emergence of *Pax6* in Bilateria, *Rax* and *Pax6* began to act jointly in the eye development of Bilateria. Future evolutionary analyses of other homeobox transcription factors involved in eye formation, including Six3, Six6, and Lhx2, may deepen our understanding of the evolution of the eye [15].

The previous study examining *Rax* evolution analyzed earlier versions of the lamprey genome assemblies [43, 44] and identified one *Rax* gene [21]. They suggested that future studies may identify another *Rax* gene because of the incomplete nature of these genome assemblies [21]. The current study analyzed the latest assembly of the lamprey [45] and hagfish (GCA_900186335.2) genomes and identified one *Rax* gene in both genomes (Table 1). Moreover, our synteny analysis results suggest that the lamprey and hagfish *Rax* loci are orthologous (Fig. 2). Taken together, the current results further support the possibility that jawless vertebrates only possess one *Rax*.

Based on the results of our synteny and molecular phylogenetic analyses, we propose an alternative origin hypothesis of *Rax* and *Rax2 *in jawed vertebrates. These two genes might have resulted from segmental duplication of a small region containing *Malt1*, *Rax*, and *Cplx4* ancestor genes in the common ancestor of jawed vertebrates (Fig. S3). This conclusion conflicts with the previous hypothesis that vertebrate *Rax* and *Rax2* resulted from two rounds of whole‐genome duplication (WGD) [21]. Following two rounds of WGD that occurred at the root of vertebrates, many vertebrate genes have two to four paralogs [46]. For example, vertebrate genomes contain four Hox gene clusters or three Otx family genes: *Otx2*, *Crx*, and *Otx5* [47]. Likewise, the previous study used molecular phylogenetic and synteny analyses to conclude that vertebrate *Rax* and *Rax2* originated from two rounds of WGD that occurred in the common ancestor of vertebrates [21]. The authors conducted phylogenetic analysis including jawed vertebrate Rax, jawed vertebrate Rax2, and lamprey Rax. However, they did not include invertebrate Rax as an outgroup. Therefore, lamprey Rax can be arbitrarily assigned to Rax or Rax2. The authors assigned the lamprey Rax to Rax2 without detailing their justification [21]. Further, synteny analysis showed that the *Rax* and *Rax2* loci were mapped to the same regions of the lancelet genome [21]. The authors used their synteny analysis results to support the conclusion that *Rax* and *Rax2* originated from WGDs. However, segmental duplication of the ancient *Rax* locus could produce similar synteny analysis results (Fig. S4). Segmental duplications in vertebrate genomes are commonly observed. For example, ~ 4% of the human genome is covered by duplications, with segmental duplication accounting for up to 14% in individual chromosomes [48]. Segmental duplication is believed to occur via nonallelic homologous recombination in regions flanked by highly homologous sequences [49]. Since it is very likely that the genomes of jawed and jawless vertebrates’ common ancestor contained highly homologous duplicated sequences, such as transposable elements, segmental duplication events could occur frequently in their genome. Together, these considerations indicate that the synteny analysis results alone cannot completely exclude the possibility of segmental duplication. Moreover, we included four invertebrate Rax sequences as outgroups and used the lamprey and hagfish Rax sequences from their latest genomes [45] to perform more robust phylogenetic analyses than the previous study [21]. The current molecular phylogenetic analysis indicated that lamprey and hagfish *Rax* forms a sister group with *Rax* and *Rax2 *in jawed vertebrates, indicating that jawed vertebrate *Rax* and *Rax2* originated from lineage‐specific segmental duplication events, not the WGDs (Fig. 4, Figs S1 and S2). However, since it is known that jawless vertebrates show amino acid composition biases, resolving orthology among jawless vertebrate Rax and jawed vertebrate Rax and Rax2 is challenging [50]. Therefore, it should be noted that our phylogenetic analysis results cannot exclude the possibility that Rax and Rax2 generation in jawed and jawless vertebrates is due to the two rounds of WGD as proposed in the previous study [21].

Another possible explanation for the current molecular phylogenetic analysis results regarding Rax and Rax2 of jawed vertebrates and Rax of jawless vertebrates is a delayed rediploidization after genome duplication [51]. In this model, following WGD, speciation predates rediploidization [51]. This leads to independent ohnolog divergence in sister lineages that share a common WGD event and provides ohnologs solely available for lineage‐specific adaptation [51]. It has been reported that 27.1% of ohnologs showed delayed rediploidization in salmonid fish [51]. Resolving orthology is difficult if ohnologs of interest independently diverged in sister lineages that share a common WGD. Therefore, it should be noted that the current molecular phylogenetic analysis results can also be affected by delayed rediploidization.

A comprehensive ortholog search for *Rax* and *Rax2* in 86 mammalian genomes from all four major mammal groups found at least five independent *Rax2* gene loss events (Fig. 6). These comprehensive analyses enabled us to raise an alternative explanation regarding the *Rax2* loss in lagomorph and rodent (Glires). The previous study proposed that *Rax2* was lost in a common ancestor of lagomorph and rodent [21]. However, they did not show the presence of *Rax2* in squirrel species, which are a sister group to other rodent species [21]. In contrast, the current study demonstrated that *Rax2* is present in the thirteen‐lined ground squirrel, arctic ground squirrel, and yellow‐bellied marmot (Fig. 6B). This finding suggests that Lagomorpha and Rodent independently lost *Rax2*.

We observed a loss of the *Rax2* octapeptide in tetrapods and five independent *Rax2* gene loss events in mammals (Figs 5 and 6). In contrast, no *Rax* gene loss events were identified in the analyzed mammalian genomes, suggesting that *Rax* is a highly evolutionarily conserved and functionally significant gene in mammals. *Rax* gene loss events may not be present in the analyzed mammalian species because it plays an essential role in central nervous system development [8]. Deletion of the *Rax* gene in mice, which lack the *Rax2* gene, results in severe brain malformation, such as the absence of the ventral forebrain and failure of the optic vesicle to form [8]. Conversely, *Rax* can functionally compensate for loss of mammalian *Rax2* in mice [6, 7, 11, 12]. In humans, unlike *RAX2*, *RAX* mutations are associated with symptoms affecting the whole eye. *RAX* mutations result in microphthalmia [9, 10], whereas *RAX2* mutations are associated with cone–rod dystrophy or age‐related macular degeneration [52, 53]. Since *RAX2* mutations can lead to age‐related disease, we hypothesize that the effects of *RAX2* loss in some mammal lineages occur after the age of sexual maturity. Therefore, *RAX2* loss has little effect on fitness. However, it also should be noted that sudden *RAX2* loss cannot be compensated by *RAX*, as indicated by the association of *RAX2* mutations with human diseases [52, 53]. Evolutionary deletion of *Rax2* from mammal genomes might require gradual accumulation of amino acid substitutions. We found that the average Ka/Ks ratio of mammalian *Rax2* was ~ 67% greater than that of mammalian *Rax* (Fig. 7B). This difference between mammalian *Rax* and *Rax2* might partially explain why *Rax2* is more defect‐prone than *Rax* in this animal group.

## Conflict of interest

The authors declare no conflict of interest.

## Author contributions

TK and TF designed the study, performed molecular evolutionary analyses, and prepared the manuscript.

## Supporting information


**Fig. S1.** Maximum‐likelihood trees of Rax and Rax2 in various animal species (related to Fig. 4).
**Fig. S2.** Neighbor‐joining trees of Rax and Rax2 in various animal species (related to Fig. 4).
**Fig. S3.** A hypothetical model of the origin of Rax and Rax2 in jawed vertebrates.
**Fig. S4.** Two possible Rax evolution scenarios.
