# Supplementary material for: Origin and evolution of the Rax homeobox gene by comprehensive evolutionary analysis
Source: FEBS Open Bio. 2020 Mar 19;10(4):657–73. doi: 10.1002/2211-5463.12832 (PMC7137802; doi:10.1002/2211-5463.12832)
Supplement: Supplementary file 1 — Fig. S1. Maximum‐likelihood trees of Rax and Rax2 in various animal species (related to Fig. 4). Fig. S2. Neighbor‐joining trees of Rax and Rax2 in various animal species (related to Fig. 4). Fig. S3. A hypothetical model of the origin of Rax and Rax2 in jawed vertebrates. Fig. S4. Two possible Rax evolution scenarios. [file FEB4-10-657-s001.docx]

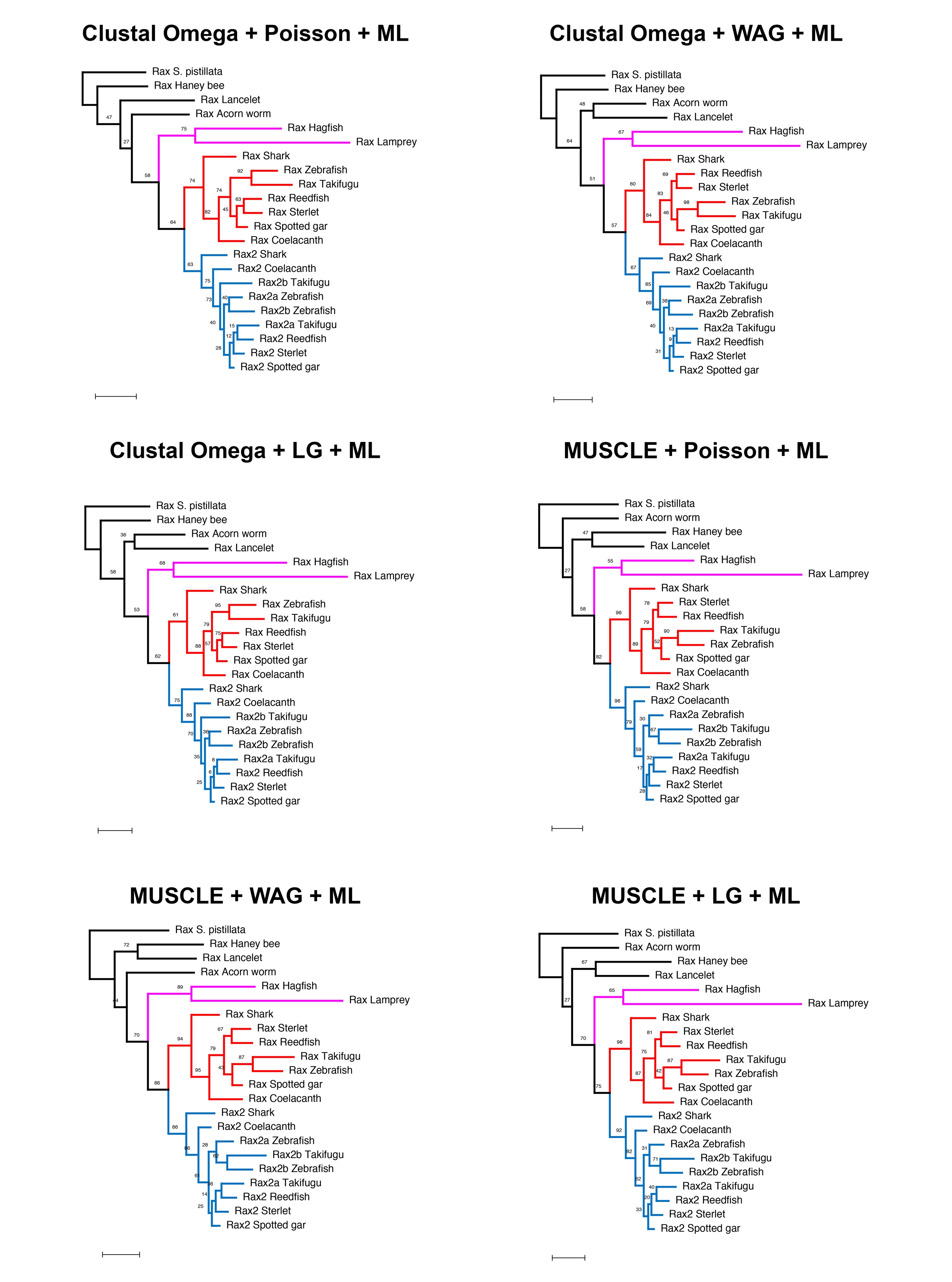


**Supplementary Figure 1. Maximum-likelihood trees of Rax and Rax2 in various animal species (related to Fig. 4)**

Maximum-likelihood trees of Rax and Rax2 in various animal species were generated using Clustal Omega and MUSCLE multiple alignment methods. The Poisson, WAG, and LG models were used for amino acid substitutions. Jawed vertebrate Rax sequences are colored in red. Jawed vertebrate Rax2 sequences are colored in blue. Lamprey and hagfish Rax sequences are colored in magenta. The scale bars represent 0.2 amino acid substitutions per site. Bootstrap values are given on each node.


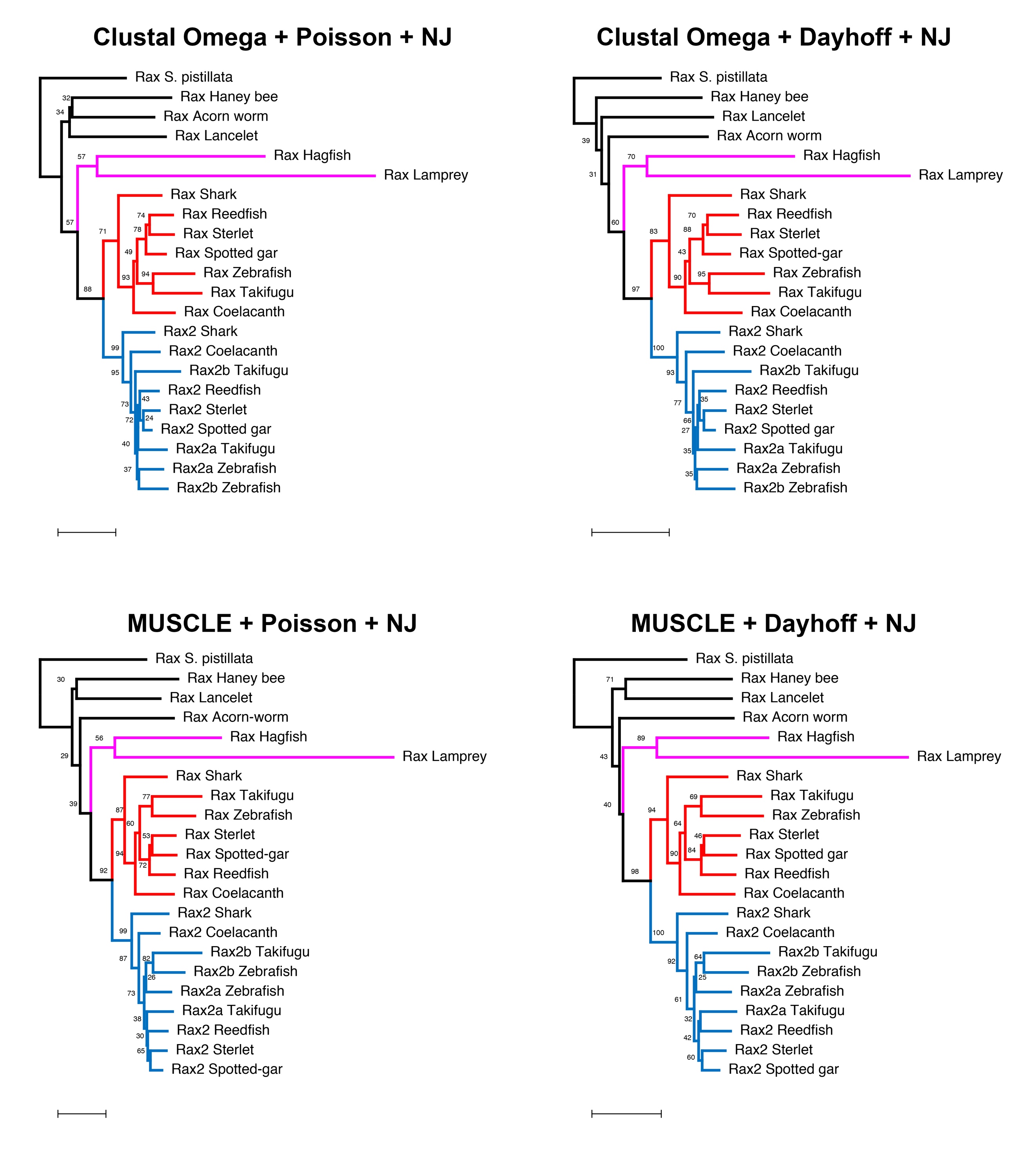


**Supplementary Figure 2. Neighbor-joining trees of Rax and Rax2 in various animal species (related to Fig. 4)**

Neighbor-joining trees of Rax and Rax2 in various animal species were generated using Clustal Omega and MUSCLE multiple alignment methods. The Poisson and Dayhoff models were used for amino acid substitutions. Jawed vertebrate Rax sequences are colored in red. Jawed vertebrate Rax2 sequences are colored in blue. Lamprey and hagfish Rax sequences are colored in magenta. The scale bars represent 0.2 amino acid substitutions per site. Bootstrap values are given on each node.

**
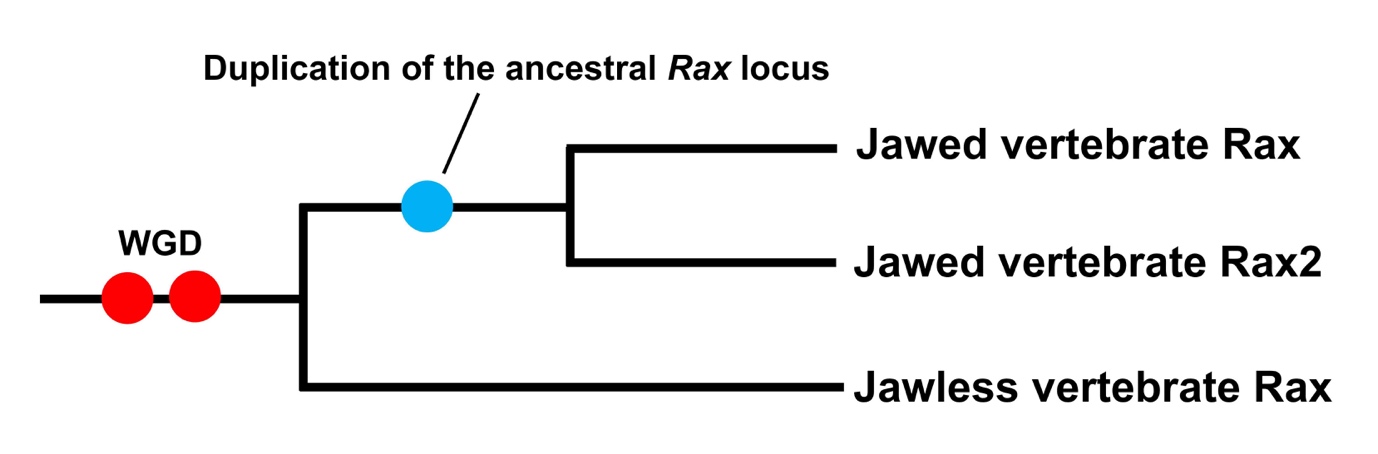
**

**Supplementary Figure 3. A hypothetical model of the origin of *Rax* and *Rax2* in jawed vertebrates**

After the two rounds of whole-genome duplication (WGD) occurred at the root of vertebrates (red circles), jawed vertebrates diverged from jawless vertebrates. *Rax2* might have appeared as a result of a segmental duplication of a small region containing the ancestors of *Malt1*, *Rax*, and *Cplx4* genes in the common ancestor of jawed vertebrates (blue circle).


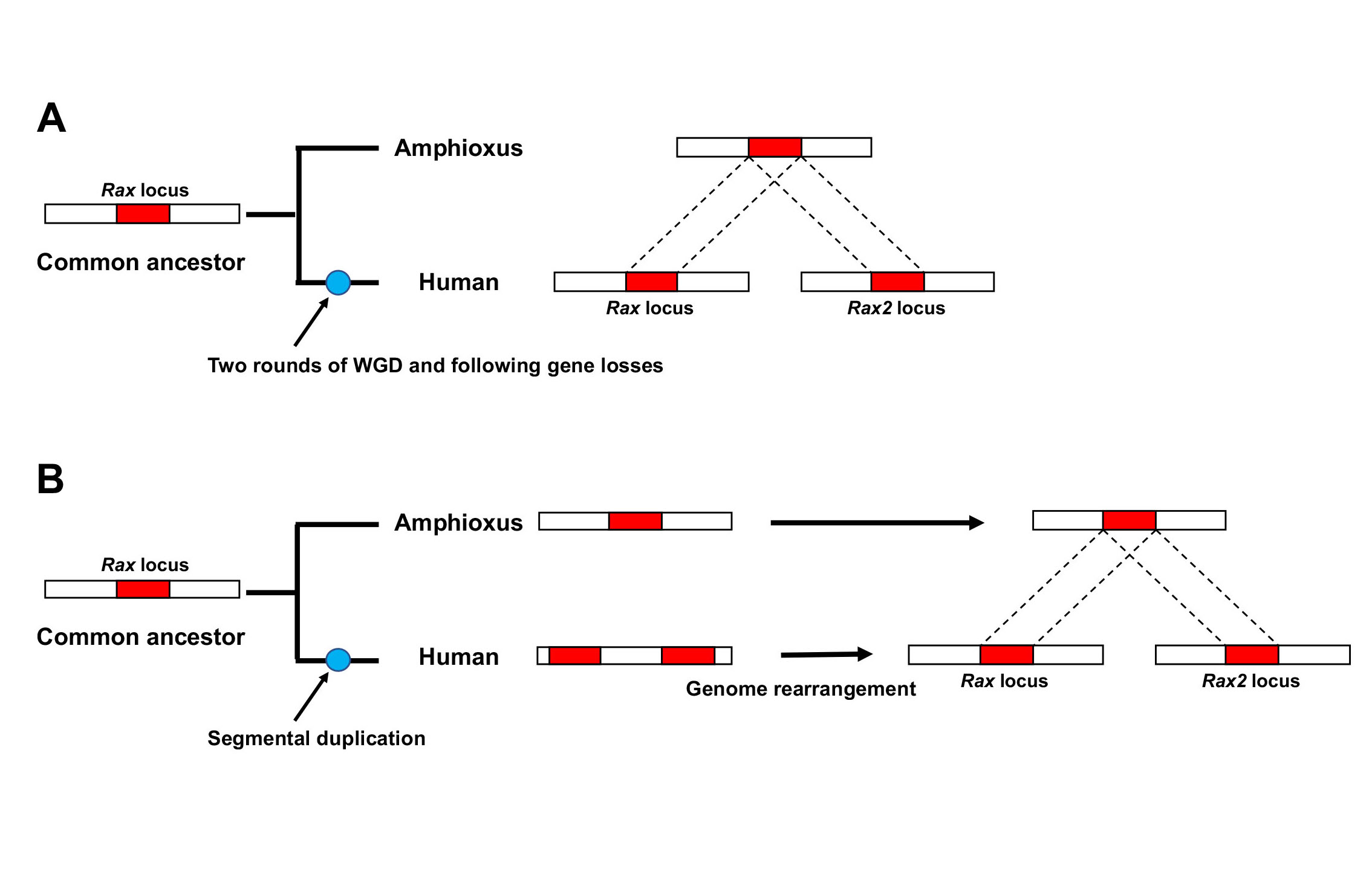


**Supplementary Figure 4. Two possible *Rax* evolution scenarios**

(**A**) Two rounds of WGD and its subsequent gene loss. In this scenario, the ancestral *Rax* locus (red box) was duplicated by two rounds of WGD (blue circle), resulting in the production of four *Rax* loci. Two *Rax* loci were then lost. As a result, two paralogous *Rax* loci (*Rax* and *Rax2*) remain in the current human genome. They should be mapped to the same region in the amphioxus genome (dashed lines).

(**B**) Segmental duplication and its subsequent genome rearrangement. In this scenario, the ancestral *Rax* locus was duplicated by segmental duplication, resulting in the generation of two *Rax* loci (blue circle). Subsequent genome rearrangement segregated the two *Rax* loci into genomic regions. As a result, two paralogous *Rax* loci (*Rax* and *Rax2* locus) remain in the current human genome. As in scenario (**A**), these two loci should be mapped to the same region in the amphioxus genome (dashed lines).
